# Tuberculous constrictive pericarditis with concurrent active pulmonary tuberculous infection: a case report

**DOI:** 10.1186/1757-1626-2-7010

**Published:** 2009-05-18

**Authors:** Yen-Wen Liu, Huey-Ru Tsai, Wen-Huang Li, Li-Jen Lin, Jyh-Hong Chen

**Affiliations:** 1 Department of Internal Medicine, National Cheng Kung University Hospital138, Sheng-Li Road, TainanTaiwan; 2 Division of Cardiology, Department of Internal Medicine, National Cheng Kung University Hospital Dou-Liou Branch345, Chuang-Ching Road, Dou-Liou, Yun-Lin CountyTaiwan; 3 Department of Cardiology, Dalin Tzu Chi General Hospital2, Min-Sheng Road, Dalin, Chia-Yi CountyTaiwan

## Abstract

**Introduction:**

In some particular endemic area, it is not uncommon to see patients with tuberculosis pericarditis. However, it takes a period of time from tuberculous pericarditis to constrictive pericarditis. There is still no report of tuberculous constrictive pericarditis concurrent with active pulmonary TB infection in a patient without previous pulmonary TB infection history. Therefore, we reported a TB constrictive pericarditis with rare disease progress.

**Case presentation:**

We report the case of a 63-year-old Taiwanese man with tuberculous constrictive pericarditis concurrent with active pulmonary tuberculous infection presenting with progressive extremities edema, puffy face, abdominal distension and dyspnea on exertion found to be caused by right heart failure. The patient was cured by pericardial stripping and anti-tuberculosis chemotherapy. We reviewed other cases of tuberculous constrictive pericarditis from the literature and described the peculiarities of this case.

**Conclusions:**

Rapid diagnosis and treatment of constrictive pericarditis are crucial to reduce mortality. In some endemic areas, *Mycobacterium tuberculosis* infection should be taken into consideration during diagnostic evaluations for constrictive pericarditis. Surgical intervention is still the treatment of choice when the patient has the symptoms or signs of pericardial constriction and right heart failure. Our case is a constant reminder that active *Mycobacterium tuberculosis* infection does present itself with uncommon presentations.

## Introduction

Constrictive pericarditis is a process of chronic fibrous thickening of the pericardium, which is frequently accompanied with calcification and prevents the diastolic filling of the heart, reducing venous return and lowering output [1,2]. Pericardial constriction is resulted from the chronic inflammation of the pericardium, which leads to pericardial scarring, thickening, fibrosis, and calcification [3]. Many etiologies have been identified, such as idiopathic chronic pericarditis, infection, after cardiac surgery, and mediastinal radiotherapy [2,4,5].

*Mycobaterium tuberculosis* is the most common cause of constrictive pericarditis in endemic area [6]. The definite diagnosis is based on one of the following criteria: (1) positive Mycobaterium tuberculosis culture from pericardial effusion or tissue, (2) positive acid-fast stain bacilli or typical caseous granuloma on pericardial biopsy specimen, or (3) positive tuberculosis polymerase chain reaction in the pericardial biopsy specimen [7].

## Case presentation

**Figure 1.**
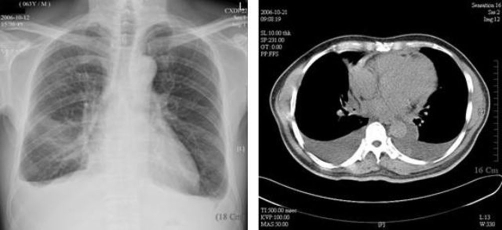
Image studies of the patient. **(A)** A chest X-ray; **(B)** a CT scan of the thorax showing thickened pericardium with areas of calcification, and bilateral pleural effusions.

A 63-year-old Taiwanese male patient was presented in October 2006 with progressive extremities edema, puffy face, abdominal distension and dyspnea on exertion for 2 weeks. There was no chest discomfort, orthopnea or paroxysmal nocturnal dyspnea. He did not have symptoms of cough, fever, foamy urine, or body weight loss. He had diabetes mellitus for years, which was well controlled with insulin. Physical examination revealed generalized pitting edema with elevated jugular venous pressure, positive Kussmaul's sign, and hepatomegaly. Bilateral pleural effusion, patchy infiltration at right middle lung field, and cardiomegaly were noted on chest X-ray (Figure 1A). Pneumonia was diagnosed by the chest specialist first in our out-patient department, and the patient was transferred to the emergency department (ED) due to the diagnosis of pneumonia and severe dyspnea. Thoracentesis, done in ED, revealed a transudate as the level of total protein and lactate dehydrogenase (LDH) of the pleural fluid were 2.800 mg/dL and 79 mg/dL, respectively, while serum protein was 7.700 mg/dL and serum LDH was 190 mg/dL.

Chest computed tomography scan also revealed thickened pericardium, up to 3 mm in diameter, minimal amount of pericardial effusion, a patchy fibrotic infiltration with bronchiectasis over the right upper lung, irregular thickening of the right intermediate bronchus, and obstruction of the right middle lung bronchus with consolidation and atelectasis of right middle lobe (Figure 1B). There were several lymph nodes, up to 2.2 cm in size, in right paratracheal, pretracheal, subcarinal and left prevascular areas.

**Figure 2.**
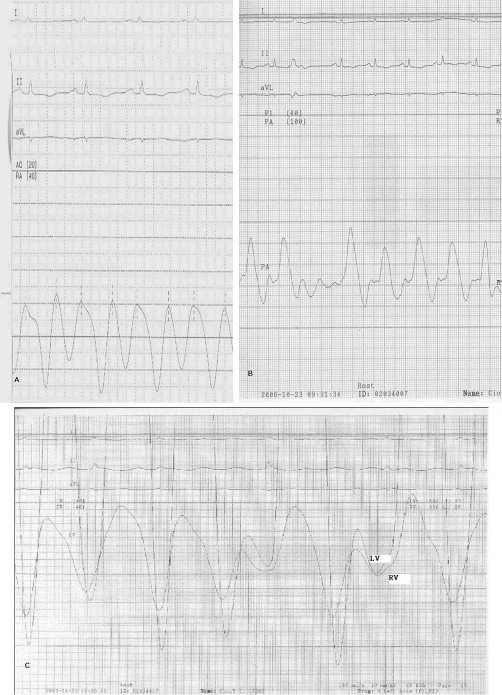
Pressure tracing of right-sided heart catheterization. **(A)** Right atrial pressure tracing; **(B)** right ventricular pressure; **(C)** simultaneous ventricular pressure tracing.

After treatment with diuretics, the peripheral edema improved significantly, but the patient still complained of severe dyspnea on exertion during the hospitalization. Echocardiograms showed thickened pericardium, especially over the right atrium, and small amount of pericardial effusion. The left ventricular wall motion was normal. However, there were limited right atrial wall motion and distended inferior vena cava with blunted respiratory variation. Under the impression of right heart failure due to constrictive pericarditis, the patient underwent cardiac catheterization. The hemodynamic studies (Figure 2A,2B, and 2C) showed right atrium pressure 24/22 mmHg, pulmonary capillary wedge pressure 23 mmHg, prominent *x* and *y* descent in right atrial pressure tracings, dip-and-plateau pattern in the right ventricular diastolic pressure tracings. There was equalization of the end-diastolic pressures between right and left ventricles (24 mmHg).

Because of the diagnosis of constrictive pericarditis complicated with right heart failure, surgical operation for pericardial stripping was performed. Operative findings included markedly thickened pericardium and enlarged mediastinal lymph nodes. The postoperative course was uneventful with improvement of heart function. Pathology of the removed pericardium showed obvious granulomatous changes with caseous necrosis.

Only one sample of sputum specimen was collected on the operation day with vigorous humidifier using normal saline for several days, which was positive for acid-fast stain. The sputum culture gave positive result of *Mycobacterium tuberculosis* four weeks later. The patient was put on quadruple anti-tuberculosis chemotherapy and remains free from symptoms of heart failure.

## Discussion

Persistent tuberculous pericarditis will lead to pericardial constriction due to chronic granulomatous inflammation of the pericardium with subsequent deposition of fibrinous strands, demonstrable on echocardiography [8]. Use of corticosteroid as an adjuvant therapy has been recommended for patients with tuberculous pericarditis and in the presence of large amount of pericardial or pleural effusion to suppress inflammation and reduce effusion. However, corticosteroids may aggravate the immunity of a compromised host, especially in the elderly [4,9,10]. Thus, the benefit of corticosteroids as adjuvant therapy in tuberculous pericarditis remains uncertain. Corticosteroids were not used in this patient, because only a small amount of pericardial effusion was detected by echocardiography initially and during follow-up period after operation. Moreover, anti-tuberculosis chemotherapy is still the cornerstone of the treatment for tuberculous pericarditis. In the endemic area, six-month anti-tuberculosis chemotherapy is recommended. The initial phase should consist of 2 months of isoniazid, rifampicin, pyrazinamide, and ethambutol. The preferred continuation phase consists of isoniazid and rifampicin given for 4 months [11].

In the chronic stage of tuberculous constrictive pericarditis, pericardial decortication with wide resection of both the visceral and the parietal pericardium, remains the definitive treatment [2]. However, there are no clear-cut determinants for surgical intervention when managed at the early stage. Yang et al. once reported that decision largely depended on the clinical symptoms of cardiac temponade, progression of heart failure, and constriction that lead to jugular vein engrossment [12]. They highlighted the importance of pursuing early pericardiectomy, rather than pericardiocentesis and window placement, to achieve sustained relief of symptoms in patients with advanced stage disease.

The diagnosis of tuberculous constrictive pericarditis in the current patient was confirmed by pathological findings. The patient did not suffer from any airway symptoms, even though atypical, for pulmonary tuberculosis, but was later verified by a positive sputum acid-fast stain and culture of *Mycobacterium tuberculosis*. It was not uncommon to see patients with tuberculous pericarditis, either effusive or constrictive, especially in endemic area, but our past experiences were that tuberculous constrictive pericarditis occurred much later after active tuberculous pulmonary infection or ineffective therapy of the pulmonary infection. There was no reported case of tuberculous constrictive pericarditis with concurrent active pulmonary tuberculosis. Our case is a constant reminder that active *Mycobacterium tuberculosis* infection does present itself with uncommon presentations.

## Conclusion

Rapid diagnosis and treatment of constrictive pericarditis are crucial to reduce mortality. In some endemic areas, *Mycobacterium tuberculosis* infection should be taken into consideration during diagnostic evaluations for constrictive pericarditis. Surgical intervention is still the treatment of choice when the patient has the symptoms or signs of pericardial constriction and right heart failure.

## List of abbreviations

ED: emergency department
LDH: lactate dehydrogenase
